# Dentistry in California

**Published:** 1861-12

**Authors:** J. D. Anderson


### Correspondence.

### DENTISTRY IN CALIFORNIA.

Dr. Taft, *Editor of Dental Register*:—As a graduate of the Ohio Dental College, I, of course, feel an interest in those who sustain the institution, and also in the Dental Register, and most willingly comply with your request by writing a communication for said journal, to give my old friends in Cincinnati, and the profession generally, an idea of dentistry in California.

A few years ago, any imposter, with a few old-fashioned pluggers and a turnkey, could, on a pedestrian journey thro' the country and in the city, from house to house, receive great encouragement in his quackery. But that time has passed, and to obtain a *permanent and respectable* practice here now, a dentist must be as well qualified as he is required to be in any other part of the country. I suppose that in no part of the United—or now "discordant and belligerent"—

States have the people suffered more from charlatanry in the dental profession than in California; and as time has revealed this to the victims, the whole fraternity has been brought into disrepute, or at least every member of it in the State is subject to suspicion until he has furnished abundant evidence of his professional skill and personal honesty. Consequently, there is not much itinerant practice done now; but the profession is by no means purged of empiricism, and all classes of dentists are here to be found in abundance. Dentists are established in nearly all the little towns in the State and in the cities are hosts of them. Some, relying upon their qualifications, maintain a professional dignity, command respect, and obtain a fair remuneration for their skillful operations—others, wonderfully indignant that their professional brethren should exact such *exorbitant* fees for services rendered the *poor laboring people*, advertise to do work *so* cheap that "the wayfaring man, though a fool," may be able to patronize them.

These same philanthropic gentlemen do not wish to make money; they only want a living, and finding their *labor of love* might not be well considered or appreciated, are so remarkably kind as to let the people know it in the streets, to visit their dwellings and tell them of it; and it is really believed by some that they "do as much work" out of the office to get "custom," as they do in the office to get clear of it.

A great many good dentists have come to this State within the last few years, who, being unable to meet the heavy expenses to which they, *ex necessitate*, were subjected, were compelled—unless they descended to the unprofessional conduct of some of their competitors—to get into some other business or to return to "the States;" and if they were enabled to continue in the practice by accepting the gauntlet of empirics by operating as cheap, and being as unprofessional, they never can accomplish anything—for, being so poorly paid, they do not feel justified in bestowing the necessary attention on their operations, or in using the right *quality*

*and quantity* of material, and consequently never acquire a desirable reputation, and nobody ever knew a "cheap dentist" to get rich. Dentists here, I am sorry to say, do not fraternize, but seem to regard each other as enemies, and consequently, there are no societies for mutual edification, and for the advancement and promotion of the profession. There are many first class dentists in the State, however, who keep thorougbly posted up, and every style of artificial teeth used in Cincinnati can be had here—not even excepting the porcelain base. There is also another style of work—the use of which is confined to the office of Thomas & Anderson, in this city—invented by Dr. W. H. Thomas, with whom I am associated—that has not yet been heard of in "the States," to my knowledge. A plate of platina is fitted to the mouth, and a combination of metals which does not tarnish, and which seems free from galvanic action, is used to unite the teeth to the plate and surround them (as the vulcanite teeth are encased in the rubber)—thus making the piece cleanly and durable. He has also been using the same style of work with cheaper materials for temporary work; but of that, together with the *modus operandi*, you may hear more anon. In my next I will write on some branch of dentistry, which I hope will be more interesting. Respectfully, yours,

J. D. ANDERSON.

